# Postpartum superior sagittal, left transverse sinus and right internal jugular vein thrombosis with left parietal infarct: A case report

**DOI:** 10.1016/j.radcr.2022.03.037

**Published:** 2022-04-05

**Authors:** Muhammad Naveed Ur Rehman, Dr. Ubaid Khan, Ayesha Aslam

**Affiliations:** a Department of Medicine, King Edward Medical University Lahore, Pakistan; b Department of Neurology, Mayo Hospital, Lahore, Pakistan

**Keywords:** Cerebral venous sinus thrombosis, Cerebral infarction, Postpartum thrombosis, Superior sagittal, Transverse sinus thrombosis, Internal jugular vein

## Abstract

Cerebral venous sinus thrombosis is a rarely occurring condition. Pregnancy and postpartum are both known risk factors for cerebral venous sinus thrombosis. Early detection and treatment are critical, as CVST can be potentially life-threatening. Here, we present a case of a patient who developed left transverse and superior sagittal sinus thrombosis 15 days after normal vaginal delivery. The patient presented to the emergency department with complaints of irritability and an altered state of consciousness for two days. The patient also developed seizures extending from the lower limb to the upper body. Laboratory investigations revealed abnormalities in the complete blood count report and urine complete examination. The patient's coagulation profile was totally abnormal, indicating a presence of a thrombus. All the other diagnostic techniques, including Electrocardiogram, Carotid Doppler Scan, and Ultrasound abdomen, revealed no findings. However, Magnetic resonance venography + Magnetic resonance imaging showed partial superior sagittal sinus thrombosis in the anterior and upper parietal regions, right internal jugular vein thrombosis, and left transverse thrombosis with associated left parietal infarcts. The presence of thrombosis in sinuses and jugular vein resulted in seizures, altered state of consciousness, and other associated symptoms. The patient was treated with sodium valproate, heparin, and other medications accordingly. The above-mentioned case was unique due to the involvement of unusual sinuses (transverse sinus) as previous studies have only reported cases of thrombus presence in the superior sagittal sinus. This case study will discuss patient diagnosis and management with Heparin and Diazepam to stop altered state of consciousness and seizures in females.

## Introduction

Cerebral venous sinus thrombosis is a rarely occurring complication of the peripartum [1]. The estimated yearly incidence of CVST is 5 per million, accounting for 0.5%-1.0% of all strokes [2]. According to the reports, in the United States, over 20000 people suffer from CVST, and associated comorbidities, including lifestyle restrictions, cognitive impairments, and dependency [3,4]. Women have a three-fold increased risk of CVST than men, and approximately 10-12 per 1,000,000 people are affected by CVST per year [5]. CVST is more prevalent in women due to specific risk factors such as oral contraception use, puerperium, pregnancy, hormone replacement therapy, cesarean section, anemia, low cerebrospinal fluid pressure due to Dural puncture, dehydration, increased homocysteine concentrations, and traumatic delivery [6,7]. In a retrospective analysis of 113 patients, can't et al. observed that 59% of individuals presented with CVST during puerperium or pregnancy [8]. CVST complicates approximately 0.004%-0.01% of pregnancies. Generally, pregnant women with CVST show symptoms such as headache, seizures, and other neurologic abnormalities. These symptoms occur as a result of blockage of the cerebral venous sinuses and impaired cerebrospinal fluid drainage, which results in intracranial hypertension. It is critical to diagnose and treat CVST quickly because it is potentially life-threatening. The following case report describes a patient who presented with an altered state of consciousness and seizures and later was diagnosed with postpartum superior sagittal and left transverse sinus thrombosis with left parietal infarct.

## Case report

A 35-year-old diabetic, hypertensive female, primigravida, presented to the emergency department with complaints of altered state of consciousness and irritability of 2-day duration. On presentation, her blood pressure, body temperature, respiratory rate, and heart rate were 140/77 mmHg, 37°C, 18 breaths/ min, 92 beats/min, respectively. Her blood sugar levels were found to be abnormally high, 397 mg/dl. Her Glasgow Glaucoma scale score was 10 of 15. She had no significant past medical or surgical history, and she, particularly, did not have a personal or family history of hypercoagulability or other hematological disorders. However, she delivered a stillbirth baby 15 days ago, otherwise, his overall pregnancy was uneventful. There was no noticeable neck stiffness and Kernig's sign and Brudzinski's sign, all were negative. The plantar response was equivocal, with the right planters going upwards and the left planters going downwards. There was spontaneous eye-opening, with bilateral normal direct and indirect light reflex. Additionally, there was no sensory deficit or cranial nerve abnormalities. Auscultation revealed normal first and second heart sounds. No added heart sounds were noted. Normal vesicular breathing was normal, and there were no added breathing sounds. No hepatomegaly or splenomegaly was observed. During her stay at the hospital, continuous blood sugar level monitoring was done.

On the second day of her admission, she also developed right-sided muscle weakness, generalized tonic colonic seizures starting from the lower limb and extending towards the upper body, which was controlled by intravenous administration of diazepam. On examination, the abdomen was soft non-tender.

Her urine complete examination was done. As mentioned in Table 1, the urine was yellowish instead of being turbid. There were significantly higher levels of glucose and Ketone in the urine. Additionally, a few red blood cells were detected.

**Table 1:** Urine complete exam.

Color	Yellow
Turbidity	NIL
PH	6.0
Protein	NIL
Glucose	++++
Ketones	++
Bilirubin	NIL
Blood	+++
Red blood cells	6 to 8
Crystal	NIL
Casts	NIL

Calcium testing revealed reduced calcium levels, as shown in Table 2. In comparison, phosphate levels were within the normal limits. Table 3

**Table 2:** Cal/ Po4.

Calcium	6 mg/dl
Phosphorus	2.1 mg/dl

**Table 3:** Coagulation profile.

PT (Prothrombin Time)	13 sec
INR (International Normalized Ratio	1.0
APTT (Activated Partial thromboplastin)	26 sec

All complete blood count findings were found to be abnormal except TLC and MCHC. The neutrophil number was raised, while the lymphocyte number was markedly low and platelets were significantly higher. This has been mentioned in Table 4. Liver function tests and renal function tests revealed no marked abnormality. Moreover, electrolytes including sodium, potassium, and chloride were all within the normal limits. Carotid doppler scan, Electrocardiogram, Ultrasound abdomen scan, chest X-ray findings were unremarkable.

**Table 4:** Complete blood count.

Hemoglobin	10.4 mg/dl
RBC (Red blood cells count)	5.2 × 10^6^/UL
HCT (Hematocrit)	35%
MCV (Mean corpuscular volume)	66 fl
Mean corpuscular hemoglobin	20pg
Mean corpuscular hemoglobin concentration	30g/dl
TLC	9.2 × 10^3/ul
Platelet's count	543 × 10^3/ul
Lymphocytes	10%
Neutrophils	83%

**Fig. 1:**
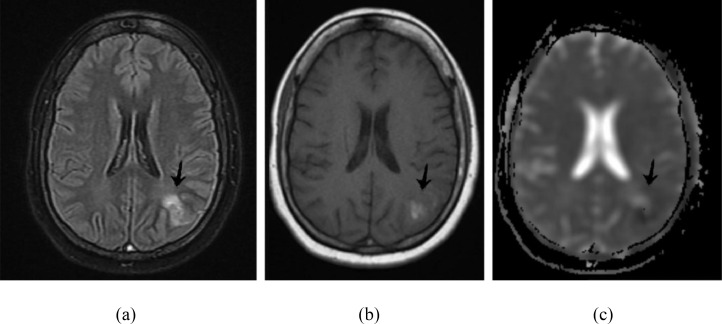
Hyperintense signals are noted in cortical and adjacent subcortical area of left parietal lobe with corresponding diffusion restriction on ADC suggestive of infarct. Note that the same area has hyperintense signals on T1 suggesting hemorrhagic nature of infarct.

**Fig. 2:**
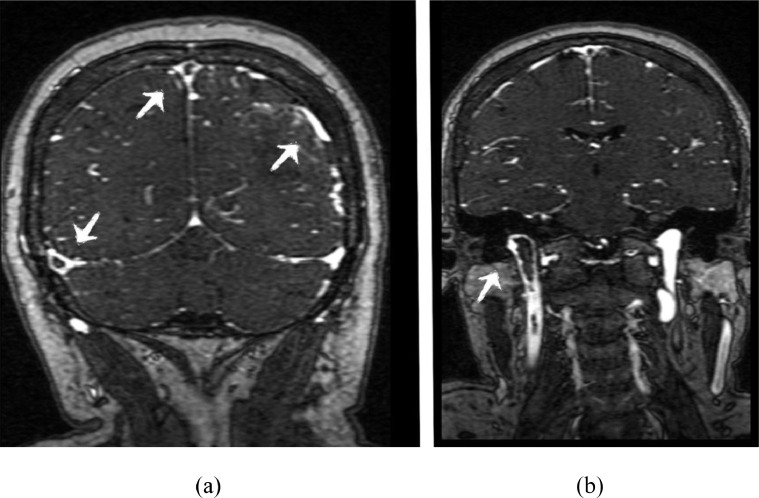
MRV-TOF coronal images showing flow voids in superior sagittal sinus, left transverse sinus and right internal jugular vein as indicated by arrow, suggestive of cerebral venous thrombosis.

Considering the patient's age, Magnetic resonance venography + Magnetic resonance imaging of the brain with contrast was performed to rule out medical conditions, including sinus thrombosis and infarctions. MRV + MRI Brain with contrast, findings revealed partial superior sagittal sinus thrombosis in the anterior and top parietal region with associated left parietal infarcts and right internal jugular vein thrombosis as shown in Fig. 1. Loss of signal void noted in left transverse sinus on T2WI/FLAIR, suggestive of left transverse thrombosis as shown in Fig. 2.

From the above-given laboratory reports and investigations, it was clear that the patient was suffering from postpartum cerebral venous sinus thrombus as MRV + MRI with contrast results revealed superior sagittal thrombosis, right internal jugular vein thrombosis, and left transverse sinus thrombosis with left parietal infarct. The patient was later transferred to the neurology ward, where she also stated her complaints about right side body weakness, eyes up rolling, and fasciculations that is signs and symptoms of upper motor neuron lesion. This verified the ultimate diagnosis as cerebral venous sinus thrombosis and associated infarction.

As a result of this diagnosis, the patient's first-line treatment strategy included intravenous injection of heparin subcutaneously twice a day, and Mannitol 150mg intravenously thrice a day, as well as blood pressure management which led to improvement in GCS score.

Overall, the treatment plan included ceftriaxone 1g intravenously twice a day, vancomycin 1g intravenously twice a day, acyclovir 750 mg intravenously thrice a day, Mgso4 1g intravenously 4 hourly and, omeprazole 40mg intravenously once a day, hydrocortisone 200mg intravenously twice a day, sodium valproate 500 mg intravenously twice a day, 6 units’ insulin thrice a day, 18 units Neutral Protamine Hagedorn insulin twice a day. The reason for IV antibiotic and IV acyclovir was the suspicion of meningoencephalitis, although signs of meningeal irritation were negative; the antibiotics were later stopped when the results of lumbar puncture came out to be negative and further diagnosis of CVST was established. Mgso4 and Mannitol administration was stopped just after 2 days. After 2 days of heparin administration, the patient was relatively stable, GCS score improved to 13 of 15. Fits were now settled; no further episode of seizure was noted. Finally, she recovered to E4 (eye-opening), V5 (verbal response) M6 (motor response) on the Glasgow coma scale and was discharged from the hospital after 17 days. As expected, the outcomes were favorable, resulting in a significant improvement in the patient's symptoms and overall condition.

## Discussion

Cerebral venous sinus thrombosis is a serious peripartum condition. According to various studies, the incidence of venous thrombosis during puerperium and pregnancy is approximately 1:10,000 to 1:25,000, and out of 10,000 deliveries, approximately 12 cases can present with CVSD [9]. CVST patients have an elevated risk of a poor outcome; it is critical to diagnose and treat the condition early and effectively. That is because sometimes, this condition is misdiagnosed due to the presence of neurological signs. The cerebral venous system is a unique system. Unlike other vessels, the major veins of the brain are made up of dural folds called sinuses. Sinuses lack muscular walls, are unable to contract, and lack valves. The sinuses not only allow for extensive venous outflow from the brain but also serve as a good location for blood to pool. Sinus thrombosis occurs when a blood clot obstructs one of the sinuses, preventing venous return [10].

Most frequently, thrombosis develops in the superior sagittal sinus [11]. But our case is a unique presentation as the patient developed superior sagittal sinus thrombosis, right internal jugular vein thrombosis, and left transverse sinus thrombosis with left parietal infarct. Risk factors for CVST include malignancy, puerperium, contraceptive use, pregnancy, and infections, for example, mastoiditis, otitis and sinusitis, cesarean section, obesity, and a history of thromboembolic disease during a previous pregnancy [12]. In our case, the major risk factor “postpartum” was identified as the patient had delivered a baby 15 days ago. According to large population-based cohort research [13], the risk of venous thromboembolism is highest during the first three weeks postpartum, and women in their third trimester face a sixfold increased risk as compared to non-pregnant women.

If CVST is predicted, then computed tomography (CT) cerebral venography or MRI venography should be performed. Additionally, CVST is difficult to detect using these traditional diagnostic techniques due to the small size of the thrombus. According to a recent study, T2 /susceptibility-weighted MRI sequences have been shown to be quite successful at detecting such lesions. In our case, the diagnosis of cerebral venous sinus thrombosis was made on the basis of MRI + MRV results which revealed superior sagittal and left transverse sinus thrombosis with left parietal infarct.

Although treatment for CVST is still debatable, heparin has been reported to be an effective and safe option. Anti-seizure medicines should be administered to individuals who exhibit early seizures. Anticoagulation therapy for CVST prevents thrombus aggravation and improves the occlusion lesion. This treatment approach is recommended by the “European Federation of Neurological Societies guidelines” [1]. However, adequate management is very contentious in cases of hemorrhagic appearance. Approximately 39%-41% of CVST patients manifest with isolated subarachnoid hemorrhage, intracerebral hemorrhage, and hemorrhagic venous infarcts. While warfarin or heparin have been used for over 50 years, newer oral anticoagulants (rivaroxaban, apixaban, or Dabigatran) may offer an alternative strategy to conventional therapy for pulmonary embolism and CVST [14]. This case demonstrates the critical importance of clinical suspicion, supporting investigations, and a high index of suspicion in establishing a diagnosis.

## Conclusion

Cerebral venous superior sagittal and left transverse sinus thrombosis is a rare, potentially fatal complication that mostly affects females in their third trimester. A female who suffers from CVST presents with a complaint of headache, development of seizures, and irritability. If it is left untreated, this condition can result in the death of the patient. Appropriate diagnosis and therapy appear to be critical for avoiding a decline in neurological function as this condition is difficult to diagnose due to the presence of misleading neurological signs. The current mainstay of treatment of CVST is the anti-seizure drugs al to prevent seizures and the use of heparin or oral anticoagulants to dissolve thrombus.

## Author's contribution

All authors contributed toward data, drafting, and revising the paper, gave final approval of the version to be published, and agree to be accountable for all aspects of the work.

## Ethical approval

For this case ethical approval was not required from hospital, and we have patient father consent form.
